# No effect of yeast-like fungi on lipid metabolism and vascular endothelial growth factor level in children and adolescents with type 1 diabetes mellitus

**DOI:** 10.1186/s13052-016-0317-9

**Published:** 2016-12-12

**Authors:** Katarzyna Zorena, Beata Kowalewska, Małgorzata Szmigiero-Kawko, Piotr Wąż, Małgorzata Myśliwiec

**Affiliations:** 1 Department of Immunobiology and Environment Microbiology, Medical University of Gdańsk, Dębinki 7, Gdańsk, 80-211 Poland; 2 Department of Tropical Medicine and Epidemiology, Institute of Maritime and Tropical Medicine, Medical University of Gdańsk, Gdańsk, Poland; 3 Clinics of Paediatrics, Diabetology and Endocrinology, Medical University of Gdańsk, Gdańsk, Poland; 4 Department of Nuclear Medicine, Medical University of Gdańsk, Gdańsk, Poland

**Keywords:** Type 1 diabetes mellitus, Children and adolescents, Lipid metabolism, Vascular endothelial growth factor, Yeast-like fungi

## Abstract

**Background:**

The objective of the research was to investigate vascular endothelial growth factor (VEGF) levels in the context of lipid metabolism and amount of yeast-like fungi colonizing the digestive tract in children and adolescents with type 1 diabetes mellitus (T1DM).

**Methods:**

The study included 45 children with T1DM and 27 age- and sex-matched healthy control subjects. In the study sample 33 T1DM patients were administered insulin pump therapy and 12 T1DM patients were administered multiple daily injections with insulin pen devices. All T1DM patients were free of micro- and macrovascular complications. In T1DM patients and healthy controls biochemical tests were performed and measurements of yeast-like fungi colonizing the alimentary tract were conducted. Moreover all study subjects had their serum VEGF levels measured with ELISA test.

**Results:**

The subgroup of children and adolescents with T1DM and yeast-like fungus colony number 10^^3^ CFU/g was shown statistically significantly lower HbA1c levels, and lower but not statistically significantly total cholesterol, LDL cholesterol and VEGF levels versus T1DM patients with the amount of yeast-like fungi 10^^6^ CFU/g. Moreover higher HDL levels were observed in this subgroup versus T1DM patients with the amount of yeast-like fungi 10^^6^ CFU/g although the difference was not statistically significant.

**Conclusions:**

Our study has shown no influence of yeast-like fungi on lipid metabolism and VEGF level in children and adolescents with T1DM. Comprehensive treatment of T1DM patients and intensive insulin therapy with help of personal insulin pumps can reduce or prevent the development of long-term diabetic complications. Further studies in this field are needed.

## Background

In view of the currently available data hyperglycaemia is known to be the main factor that contributes to the development of angiopathic complications in diabetes [1–3]. Metabolic control of diabetes mellitus is one of the most important factors, but not the only one, that affects the risk of long-term angiopathic complications [4–7]. Several proofs of evidence indicates that the risk of long-term diabetic complications is increased when hyperglycaemia is accompanied by lipid metabolism disorders and high blood pressure values [3, 6, 8]. In our previous studies we showed a correlation between systolic blood pressure values and serum levels of vascular endothelial growth factor (VEGF) in children and adolescents with type 1 diabetes mellitus (T1DM) [8]. VEGF is a potent mitogenic and chemotactic factor for endothelial cells, thereby stimulating angiogenesis. VEGF can increase vascular permeability, favour oedema and enhance migration of cells from the circulating blood into the sites of inflammation. VEGF is an important factor that triggers inflammatory processes in chronic diseases [9].

Prevention of long-term complications is among the most important treatment goals in T1DM patients. All patients with T1DM have to be monitored for HbA1c levels, lipid metabolism parameters, blood pressure and other known risk factors of long-term vascular complications [5, 10, 11]. Moreover, it must be noticed that treatment of T1DM children and adolescents has significantly changed in Poland and all over the world during the last decade [12–14]. First of all new insulin therapy technologies have been developed and implemented, such as personal insulin pumps integrated with continuous glucose monitoring systems (CGM) [14, 15]. Data from clinical trials and immunological studies point at the very important role of even minor blood glucose fluctuations in the development of both micro- and macrovascular complications [15–17].

Moreover the role of yeast-like fungi in the course of diabetes has not been fully elucidated [18–20]. Antigens found in the human body trigger mechanisms of the innate and adaptive immune reactions, closely related to each other. On one hand they can result in elimination of the causative factor, but on the other hand they can lead to systemic disorders or even death [21, 22]. Efforts are being made by our team and other researchers to investigate the yeast-like fungus virulence and its sequelae in T1DM patients [20, 21, 23]. However proving the causal relationship between infections and development of complications in T1DM patients remains a big challenge. In many cases the main problem involves a long latency time between exposure to the yeast-like fungus antigen and the occurrence of clinical signs and symptoms of long-term angiopathic complications [22]. Therefore the aim of our current study was to investigate VEGF levels in the context of selected lipid metabolism parameters and the amount of yeast-like fungi colonizing the alimentary tract of T1DM children and adolescents.

## Methods

The study involved 45 adolescent patients (16 girls and 29 boys) with T1DM. Diabetes was diagnosed according to the Polish Diabetes Association guidelines which correspond with the guidelines of the WHO [12, 24]. All T1DM patients were free of micro- and macrovascular complications. T1DM children and their parents/legal guardians were provided comprehensive care by diabetes treatment team composed of diabetologist, nurse, dietician, psychologist, physiotherapist and educator. In the study sample 33 T1DM patients were administered insulin pump therapy and 12 T1DM patients were administered multiple daily injections with insulin pen devices. Blood glucose levels were measured by continous glicemic monitoring systems (CGMS) using an electrode compatible with “Guardian” device or Medtronic insulin pump (Medtronic Minimed, Northridge, CA). Glycated haemoglobin (HbA1c) was measured with an immunoturbidometric method using a Unimate 3 set (Hoffmann-La Roche AG, Basel, Switzerland). Reference values for healthy people estimated by the local laboratory ranged from 4.3 to 5.7% (35–42 mmol/mol). The urinary albumin was measured by immunoturbidometric assay using Tina-quant kit (Boehringer Mannheim GmbH, Germany). Microalbuminuria was diagnosed when in at least two out of three urine samples daily albumin excretion was between 30 and 300 mg/24 h, collected within 6 months from patients with well controlled diabetes with no clinical or laboratory signs of ketoacidosis. Serum total cholesterol, low density lipoprotein (LDL) and high density lipoprotein (HDL) cholesterol levels were assayed by the ARCHITECT cSystem and AEROSET, Abbott, Wiesbaden, Germany. Lipid profile was assessed according to the value [25]. Blood pressure was measured using a 24-h blood pressure monitoring (ABPM) method. Various sizes of the cuff were used according to age, weight and arm circumference of the studied subjects. All the ABPM results, which had less than 80% of technically correct measurements were excluded from the study. Threshold values defining range of normal blood pressure values, pre-hypertension and hypertension state were according to the centile tables which took into the consideration the gender, age and height centile. Arterial hypertension was diagnosed when mean ABPM values were above the 95th centile for the corresponding age, gender and height on at least three separate measurements [26].

In order to enumerate yeast-like fungal colonies in 1 g faeces, quantitative cultures on Sabouraud Dextrose Agar were used. The tested materials were fresh faecal samples from T1DM children and healthy control subjects. The samples were collected to sterile containers and subjected to further test procedures. Faecal suspensions in normal saline in serial dilutions 1:10, 1:100, 1:1000, 1:10,000 were prepared and incubated for 72 h at 37 °C. According to the number of colony forming units grown in 1 g faeces the following index values for the fungus growth were established: from 0 to 10^^3^ CFU/g and from more than 10^^3^ to 10^^6^ CFU/g.

Patients with T1DM and their matched controls were examined by a pediatrician on the day of collection of the faecal samples. Medical history was taken and physical examination was performed and did not reveal any gastrointestinal complaints in either study group. Moreover the study participants had not been receiving antibiotics for up to 3 months prior participation to the study. Children with symptoms of infection or systemic somatic illness other than diabetes mellitus were excluded from the study. The study patients with T1DM were divided into two subgroups: with duration of diabetes ≤5 years and those with disease duration >5 years.

Control group consisted of 27 healthy children and adolescents, age and BMI matched (13 girls and 14 boys).

Written informed consent was obtained from all children and adolescents participating in the study, or from their parent or guardian. The study was approved by the Ethics Committee of the Medical University of Gdańsk (no NKBBN/125/2014) and the investigation was carried out in accordance with the principles of the Declaration of Helsinki as revised in 1996.

### Serum level of VEGF

Serum level of vascular endothelial growth factor was measured by immunoenzyme ELISA method (Quantikine High Sensitivity Human by R&D System, Minneapolis, Minn., USA) according to manufacturer protocol. Minimum detectable concentrations were determined by the manufacturer as 5.0 pg/ml. Intra-assay was 53.7 and inter-assay 910 precision performance of the assay were determined on 20 replicates from the quality control data of the laboratory.

### Statistical analysis

Statistical analyses were performed with the RKWard Data Analysis Tool Version 0.6.1 using the KDE Development Platform 4.13.3 [27]. The Shapiro-Wilk’s test was used to evaluate normality of variables. The differences between the groups were calculated with T Student’s or the non- parametric U Mann Whitney tests. In all analyses a two-tailed significance level <0.05 was regarded as statistically significant. A univariate and multivariate logistic forward regression analysis was used to assess the association between VEGF and clinical parameters and yeast-like fungal colonies in 1 g faeces with *p* < 0.05 for entry.

## Results

### Clinical characteristics of the study participants

The study involved 45 children and adolescent with T1DM aged 9.2 +/− 3.6 years, and 27 healthy children and adolescents, age range 9.8 +/− 4.5 years. The group of patients with T1DM showed significantly higher HbA1c levels *p* = 0.005, albumin excretion rate *p* = 0.007 and VEGF *p* = 0.001 versus control subjects. No significant differences were seen in age *p* = 0.65, BMI *p* = 0.59, total cholesterol *p* = 0.214, LDL-cholesterol *p* = 0.214 and HDL-cholesterol levels *p* = 0.098 versus healthy control subjects. Clinical characteristics of the studied patients with T1DM as well as the control healthy subjects are presented in Table 1.

**Table 1:** Clinical characteristics of patients with T1DM and healthy control subjects

	Patients with T1DM *n* = 45	Healthy control subjects *n* = 27	*p*-value
Age (years)	9.2 +/− 3.6	9.8 +/− 4.5	*p* = 0.65
Duration of diabetes (years)	5.0+/− 3.0	-----	------
HbA1c %	7.7 ± 1.24	4.2 ± 0.7	*P* = 0.005*
BMI (kg/m^2^)	21.3 ± 1.9	20.2 ± 1.4	*P* = 0.59
Albumin excretion rate (mg/24 h)	13.2 ± 7.4	3.75 ± 0.85	*p* = 0.007*
Total cholesterol (mg/dl)	176.53 ± 26.9	174.3 ± 32.7	*p* = 0.089
LDL-cholesterol (mg/dl)	103.8 ± 22.07	95.0 ± 30.6	*p* = 0.214
HDL-cholesterol (mgl/dl)	54.28 ± 11.38	58.6 ± 13.1	*p* = 0.098
Systolic blood pressure (mmHg)	113.0 ± 10.0	110.0 ± 9.0	*p* = 0.64
Diastolic blood pressure (mmHg)	70.0 ± 9.0	67.0 ± 8.0	*p* = 0.58
VEGF (pg/ml)	324.54 ± 185.61	120.45 ± 38.50	*p* = 0.001*

The results are presented as ± SD, significance (*p* <0.05)

*Abbreviations*: *T1DM* diabetes mellitus type 1, *BMI* body mass index, *LDL* Low-density lipoprotein cholesterol, *HDL* High-density lipoprotein cholesterol, *SBP* systolic blood pressure, *DBP* diastolic blood pressure, *VEGF* vascular endothelial growth factor

*Patients with T1DM vs healthy control subjects

### Clinical characteristics of T1DM patients according to their disease duration

The subgroup of T1DM patients with duration of diabetes ≤5 years was statistically significantly younger, *p* = 0.03, and achieved optimal metabolic control, *p* = 0.01, versus T1DM patients with duration of diabetes >5 years. Moreover T1DM patients with disease duration ≤5 years were found lower total cholesterol level, *p* = 0.49, lower LDL level, *p* = 0.56, and lower VEGF level, *p* = 0.37, although none of these differences was statistically significant. Patients with shorter duration of diabetes were found statistically significantly higher HDL cholesterol levels versus T1DM patients with disease duration >5 years (Table 2).

**Table 2:** Clinical characteristics of T1DM patients according to their disease duration

Duration of diabetes	Age (years)	HbA1c (%)	VEGF (pg/ml)	Total cholesterol (mg/dl)	LDL-cholesterol (mg/dl)	HDL-cholesterol (mg/dl)
<=5 years *n* = 31	10 ± 4.0	7.0 ± 1.2	316.5 ± 21.7	172.1 ± 23.4	99.1 ± 17.1	61.12 ± 8.3
>5 years *n* = 14	13 ± 2.0	8.1 ± 0.9	389.4 ± 23.8	175.2 ± 25.4	105.6 ± 20.1	53.4 ± 12.9
*p*-value	0.03*	0.01*	0.37	0.49	0.56	0.02*

The results are presented as ± SD, significance (*p* <0.05)

*Abbreviations*: *VEGF* vascular endothelial growth factor, *LDL* Low-density lipoprotein cholesterol, *HDL* High-density lipoprotein cholesterol

*Patients with < =5 years vs >5 years duration of diabetes

### Clinical characteristics of T1DM patients according to the amount of yeast-like fungi colonizing the digestive tract

The subgroup of children and adolescents with T1DM and yeast-like fungus colony number 10^^3^ CFU/g was shown statistically significantly lower HbA1c levels, *p* = 0.01, lower but not statistically significantly total cholesterol and LDL cholesterol levels and lower but not statistically significantly VEGF levels versus T1DM patients with the amount of yeast-like fungi 10^^6^ CFU/g. Moreover higher HDL levels were observed in this subgroup versus T1DM patients with the amount of yeast-like fungi 10^^6^ CFU/g, *p* = 0.41, although the difference was not statistically significant (Table 3).

**Table 3:** Clinical characteristics of T1DM patients according to the amount of yeast-like fungi colonizing the digestive tract

Yeast–like fungi	HbA1c %	Total cholesterol (mg/dl)	LDL-cholesterol (mg/dl)	HDL-cholesterol (mg/dl)	VEGF (pg/ml)
10^^3^ CFU/g *n* = 19	7.1 ± 0.8	172.81 ± 29.33	101.87 ± 23.65	59.4 ± 8.3	327.03 ± 179.9
10^^6^ CFU/g *n* = 26	7.4 ± 1.9	183.52 ± 20.89	107.41 ± 18.89	51.6 ± 12.4	370.37 ± 194.84
*p*-value	0.01*	0.14	0.23	0.41	0.32

The results are presented as ± SD, significance (*p* < 0.05)

*Abbreviations*: *VEGF* vascular endothelial growth factor, *LDL* Low-density lipoprotein cholesterol, *HDL* High-density lipoprotein cholesterol

*Patients with T1DM and yeast-like fungi 10^^3^ CFU/g vs 10^^6^ CFU/g

### Investigation of relationship between duration of diabetes and clinical parameters in T1DM patients

**Fig. 1:**
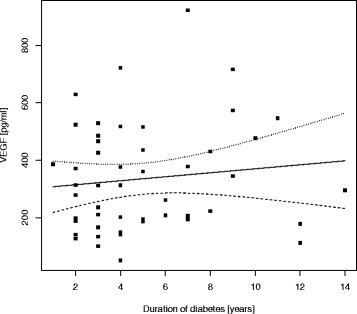
Correlation between serum VEGF level and duration of diabetes in T1DM patients

In T1DM children a positive but statistically insignificant correlation was seen between duration of diabetes and VEGF levels; Rs = 0.113, *p* = 0.44 (Fig. 1).

### Investigation of relationship between serum VEGF levels and lipid profile in T1DM children and adolescents

**Fig. 2:**
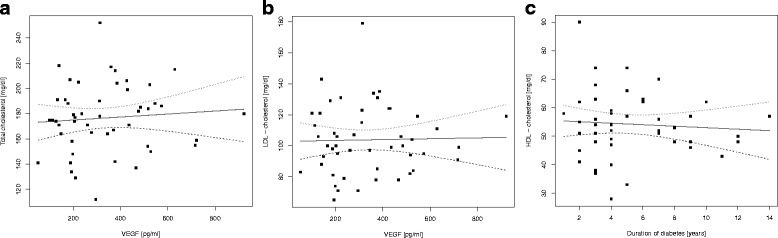
**a** Correlation between serum VEGF and total cholesterol levels in T1DM patients. **b** Correlation between serum VEGF and LDL-cholesterol levels in T1DM patients. **c** Correlation between serum VEGF and HDL-cholesterol levels in T1DM patients

In the group of T1DM children a positive but statistically insignificant correlation was seen between serum VEGF and total cholesterol levels, Rs = 0.105, *p* = 0.459 (Fig. 2a), and between VEGF and LDL cholesterol levels, Rs = 0.005, *p* = 0.973 (Fig. 2b). A negative, but statistically insignificant correlation was found between VEGF and HDL-cholesterol levels, Rs = 0.002, *p* = 0.988 (Fig. 2c).

In the next step of the research logistic regression analysis was conducted and showed no effect of HbA1c and lipid profile on VEGF levels in T1DM patients (Table 4).

**Table 4:** Logistic regression analysis results for the influence of Hb1A and lipid levels on VEGF levels in T1DM patients

DV : VEGF	β	*p*-value
HbA1c	31.761001	0.704
Total cholesterol	1.7568	0.441
LDL-cholesterol	−1.6030	0.544
HDL-cholesterol	−0.5385	0.852
Intercept	221.232	0.276

*Abbreviations*: *DV* dependent variable, *β* standard coefficient of regression, *VEGF* vascular endothelial growth factor, *LDL* Low-density lipoprotein cholesterol, *HDL* High-density lipoprotein cholesterol

## Discussion

Our previous studies and studies by other authors have shown that long-term diabetic complications occur after 5 years of the disease [1, 4, 8]. Therefore for the purpose of analyses the study patients with T1DM were divided into two subgroups: patients with duration of diabetes ≤5 years and those with disease duration >5 years. In our study the subgroup of T1DM children and adolescents with duration of diabetes ≤5 years and HbA1c level (7.0 + 1.2%) did not show any significant differences in total cholesterol and LDL cholesterol levels versus T1DM patients with disease duration >5 years and HbA1c (8.1 + 0.9%). Higher HDL cholesterol levels were seen in T1DM children with disease duration <5 years and HbA1c = 7.0 + 1.2% versus T1DM patients with disease duration >5 years and HbA1c = 8.1 + 0.9%. Currently available data on lipid metabolism in T1DM patients are not unanimous [6, 28–30]. Recently, unexpected results have been presented by *Klein* et al., who studied 730 patients for 24 years, each patient having been tested four times, and did not show any relationship between increased serum levels of oxidized low-density lipoprotein and frequency of macular oedema or diabetic retinopathy severity in T1DM patients [28]. In other studies the authors pointed at lipid profile abnormalities in patients with disease duration longer than 5 years, with poor metabolic control and cardiovascular complications [6, 29, 30].

In our study no significant differences in serum levels of VEGF were detected in patients with T1DM and duration of diabetes ≤5 years versus T1DM patients with disease duration of >5 years. In T1DM children a statistically insignificant correlation was seen between VEGF levels and duration of diabetes, total cholesterol and LDL cholesterol levels. This lack of correlation between VEGF serum levels and lipid metabolism or HbA1c levels can be partly explained by improved methods of insulin therapy in T1DM patients and partly by anti-inflammatory effects of HDL cholesterol.

Our results are in agreement with other authors who found a negative correlation between serum VEGF, CRP and HDL cholesterol levels in T1DM insulin pump patients [31]. The authors suggested that insulin pump therapy could result in considerably higher HDL cholesterol levels which could reduce both CRP and VEGF levels. The continuous insulin infusion with insulin pump in T1DM patients is likely to prevent atherosclerosis progression, thereby reducing the risk of cardiovascular disease [31]. Admittedly, our previous studies and studies conducted by other authors showed statistically significantly higher serum VEGF levels in children and adolescents with T1DM [32–35]. However the studied group of T1DM children and adolescents was older, showed poor metabolic control and had clinically overt diabetic retinopathy (DR), nephropathy and arterial hypertension [8, 32–35]. In our present study T1DM patients were free of long-term diabetic complications and had shorter history of diabetes and lower HbA1c levels.

In the end step of the study the effect of yeast-like fungi on lipid profile and VEGF levels was investigated. In the studied group of T1DM children and adolescents with the amount of yeast-like fungi 10^^3^ CFU/g significantly lower HbA1c levels were seen as well as lower total cholesterol, LDL cholesterol and lower serum VEGF levels versus T1DM patients with the amount of yeast like fungi colonizing the alimentary tract 10^^6^ CFU/g. We suggest, that in patients with a short history of diabetes of <5 years and good metabolic control as well as normal lipid profile the prevalence of the yeast-like fungi in the digestive tract is not significantly increased. This could be related to the fact that children had not complained of any gastrointestinal problems and had not been treated with any antibiotics for up to 3 months prior to their faecal sample collection. What’s more, about 75% of T1DM patients were administered insulin therapy with insulin pumps integrated with continuous glucose monitoring systems. It must be emphasized that children with T1DM are currently achieving HbA1c target levels more and more frequently thanks to the use of personal insulin pumps integrated with continuous glucose monitoring systems [13–15]. T1DM children and their parents and/or legal guardians were provided with comprehensive care educator. Education motivates patients to respond to the challenges of insulin therapy, eliminates stress and prevents anxiety about the future [10].

## Conclusions

Our study has shown no relationship between serum VEGF levels and lipid metabolism and no influence of yeast-like fungi on VEGF levels in T1DM patients. Comprehensive treatment of T1DM patients and intensive insulin therapy with help of personal insulin pumps can reduce or prevent the development of long-term diabetic complications. Further studies in this field are needed.

## Abbreviations

BMI: Body mass index
CGMS: Continous glicemic monitoring systems
DBP: Diastolic blood pressure
DR: Diabetic retinopathy
DV: Dependent variable
HDL: High-density lipoprotein cholesterol
hsCRP: high sensitivity c-reactive protein
LDL: Low-density lipoprotein cholesterol
SBP: Systolic blood pressure
T1DM: Type 1 diabetes mellitus
VEGF: Vascular endothelial growth factor
